# Work addiction and quality of life: a study with physicians

**DOI:** 10.1590/S1679-45082017AO3960

**Published:** 2017

**Authors:** Walter Fernandes de Azevedo, Lígia Andrade da Silva Telles Mathias

**Affiliations:** 1 Universidade Federal da Paraíba, João Pessoa, PB, Brazil.; 2 Faculdade de Ciências Médicas, Santa Casa de São Paulo, São Paulo, SP, Brazil.

**Keywords:** Work, Quality of life, Risk factors, Physicians

## Abstract

**Objective:**

To evaluate the quality of life of physicians and investigate to what extent it is affected by work addiction.

**Methods:**

This is an exploratory, descriptive and cross-sectional study, conducted with 1,110 physicians. For data collection, we used a questionnaire with sociodemographic information, the World Health Organization Quality of Life BREF, and the Work Addiction Scale.

**Results:**

Most physicians presented high quality of life. Female participants presented lower quality of life in the domains psychologic, environment and general (p<0.05). Quality of life was negatively correlated with the number of shifts (p<0.005). The higher the addiction to work, the lower the quality of life.

**Conclusion:**

The research allowed understanding the implications of work addiction in the quality of life. Further studies are required to support the development of strategies that improve health conditions and quality of life of medical professionals.

## RESUMO

**Objetivo:**

Avaliar a qualidade de vida de médicos e investigar em que medida a adição ao trabalho a afeta.

**Métodos:**

Trata-se de um estudo exploratório, descritivo e transversal, realizado com 1.110 médicos. Para coleta de dados, optou-se por utilizar um questionário contendo informações sociodemográficas, bem como aplicar o instrumento *World Health Organization Quality of Life-BREF* e a Escala de Adição ao Trabalho.

**Resultados:**

Os médicos, em sua maioria, apresentaram alta qualidade de vida. Os participantes do sexo feminino tiveram menor qualidade de vida em relação aos homens nos domínios psicológico, meio ambiente e geral (p<0,05). A qualidade de vida correlacionou-se negativamente com o número de plantões (p<0,005), e quanto maior a adição ao trabalho, menor a qualidade de vida.

**Conclusão:**

A pesquisa permitiu o conhecimento das implicações da adição ao trabalho sobre a qualidade de vida. Novos estudos são necessários para subsidiar a elaboração de estratégias que melhorem a saúde e a qualidade de vida do profissional médico.

## INTRODUCTION

According to the World Health Organization (WHO), quality of life (QL) involves how individuals perceives their position in life, in the context of the culture, and in the value system in which they live, in relation to their goals, expectations, standards and concerns. It is a broad concept that encompasses health in its physical and psychological instances, level of independence, social relations, personal beliefs and the environment.^1^

The scientific production on QL in Brazil is relatively recent and grows every year, without being restricted to a certain social group, but mostly covering adults suffering from some disease. This reflects the concern about understanding how these diseases compromise the life of these individuals.^2^

Research has also been conducted on the QL of health professionals and, for physicians, in particular, some studies show that stressors, such as dissatisfaction with the work environment and working conditions, as well as an excessive number of patients, can impact the QL of these professionals.^3^

Low QL in the work environment of health professionals, including physicians, may have negative consequences, such as increased absenteeism, lack of interest in activities, increased work-related accidents, apathy, muscle tension, tachycardia, headache, depression, sleep changes, as well as other physical, psychic and social problems. These impacts, in turn, may affect the individual’s motivation to work, as well as the quality of care provided to patients.^4^

Health workers with poor QL cannot properly see patients, since QL directly interferes with their working conditions and how they perform their activities.^5^

It is noteworthy that increasing demands in the labor market, based on the principle of maximum productivity, brought to the surface a new problem that can affect the QL of professionals: work addiction or workalholism.^6^

Work addiction is defined as a work-related injury, characterized by excessive and constant labor.^7^ It belongs to the class of behavioral addictions, also called non-substance addictions, and is quite frequent in our society.^8^

Workaholics usually build their lives around work, are perfectionist, deny having any health problems, and when they fall ill, they return to work before having fully recovered, which leads to physical injury and increases the risk of complications. For workaholics*,* being extremely busy is more than a lifestyle. It is a prerequisite for professional success.^9^

Work addicts may experience some physical symptoms, such as extreme tiredness, hypertension, insomnia, gastritis, alopecia, vascular and cardiac problems. However, since these symptoms alone are not proof of a possible work addiction, the correct diagnosis must also assess psychological and social aspects.^10^

A lifestyle based on workaholism can effectively lead to high levels of anxiety and depression.^11^ Moreover, this problem is associated with low satisfaction with life and impaired performance at work.^12,13^

In view of the above, there is a need to investigate the relation between work addiction and QL for physicians, since these professionals, in general, have an excessive workload, which can lead to severe health problems and, consequently, decrease their QL.

## OBJECTIVE

To evaluate the quality of life of physicians and investigate to what extent it is affected by work addiction.

## METHODS

This is an exploratory, descriptive and cross-sectional study, with a quantitative approach, performed with physicians from the State of Paraíba.

Originally, the study population consisted of 6,100 physicians registered at the Regional Medical Council (CRM-PB), who were personally contacted in hospitals and during medical meetings in the State. To define the sample, the following inclusion criteria were established: practicing during the period of data collection; interested and willing to participate in the study. A total of 1,110 physicians who met these criteria were enrolled in the investigation. The sample was calculated based on a confidence interval with a margin of error of 3% and a confidence level of 95%.

Data were collected from June to October 2015, through the application of a questionnaire with sociodemographic and professional information (gender, marital status, age, race, income and number of shifts, with a shift consisting of 12 hours of work). Data were also collected using the World Health Organization Quality of Life-BREF (WHOQOL-BREF) and the Work Addiction Scale (WAS).

The WHOQOL-BREF is a reduced version of the QL questionnaire originally developed by WHO. The current version is composed of 26 items, two of which comprise general QL evaluations; the others correspond to the items described in the original instrument, covering the physical, psychological, social and environmental domains.^14,15^ Answers to the WHOQOL-BREF questions are given on a 5-point Likert scale,^14^ with values ranging from 1 to 5. The global score and the score for each domain are calculated based on the translation of the original scale into scores ranging from zero to one hundred.^16^ The score for each WHOQOL-BREF domain is obtained on a positive scale, *i.e.,* the higher the score, the better the QL in that domain.^17^

Based on the results presented by Nunes and Freire,^18^ the 70^th^ percentile was used as the cutoff point to identify individuals with high QL. Those with scores below this percentile were classified as not having high quality of life.

The WAS, in turn, was originally developed by Schaufeli et al.^,^ and has 17 items. The Brazilian version, adapted by Carlotto et al., has the same structure and format, but it has a total of ten items, five for each dimension, answered on a 4-point Likert scale (1 for never and 4 for every day).^6^

The cutoff point for work addiction was the midpoint of the scale (2.5), *i.e.* physicians with an average score equal to or greater than 2.5 were considered work addicts. Participants with an average of less than 2.5 were not considered work addicts.

The data were originally tabulated in an Excel spreadsheet and then converted to the .sav format of the Statistical Package of the Social Sciences (SPSS), version 20. In addition to descriptive statistics (frequency distribution, percentage, mean, standard deviation and confidence interval), comparisons between means (*t* tests), the χ^2^ test and Fisher’s exact test were also carried out.

The adjustment of a logistic regression model was also considered to search for evidence regarding the influence of other variables on the occurrence of an outcome of interest of the study. To provide evidence about the quality of the adjustment made to this type of model, we used the Hosmer and Lemeshow test. We chose the stepwise method to select models with the most important variables to explain the outcome, based on a decision rule that calculates the likelihood ratio test in a given number of steps.

Regarding logistic regression, the variable classified work-addiction-related was calculated as follows: in category 1, if the physician had a score above 2.5, corresponding to a higher degree of work addiction, the variable would be rated 1. In category 2, if the score was below 2.5, implying a lower degree of work addiction, the variable would be rated zero.

This study complied with the recommendations of the National Health Council Resolution 466/12, which regulates research involving human beings. The participants signed the Informed Consent Form and data collection was initiated only after the appraisal and approval of the Human Research Ethics Committee of the *Hospital Universitário Lauro Wanderley* - *Universidade Federal da Paraíba*, under number 924.045, CAAE: 39156114.0.0000.5183.

## RESULTS

The study enrolled a total of 1,110 physicians working in the state of Paraíba, mostly men (53.30%), married (64.70%), aged between 31 and 40 (27.12%) and self-declared Caucasian (61.50%). Of the total, 38.29% worked up to ten shifts per month and 92.50% had income equal to or greater than 7 minimum wages.

Table 1 presents the statistics associated with the scores of the four QL domains and the global QL score. These scores were standardized for a scale ranging from zero to 100%.

**Table 1:** Distribution of physicians according to modified scores (scale from 0 to 100) of the domains from the quality of life questionnaire World Health Organization Quality of Life-BREF (WHOOQOL-BREF) with 26 items

WHOOQOL-BREF domains	M	SD	95%CI		p value (KS test)
			(M±1.96 *SD)		
Physical	68.9852	14.65299	40.2654	97.7051	0.000*
Psychological	67.8437	13.46486	41.4526	94.2348	0.000*
Social	67.4962	16.77387	34.6195	100.00	0.000*
Environment	60.9194	12.42355	36.5692	85.2696	0.000*
General	66.3105	11.75319	43.2742	89.3467	0.006

p value smaller than 0.00000000001 at 95% confidence interval.

M: mean; SD: standard deviation; 95%CI: 95% confidence interval.

Statistically, physicians had high QL, as expressed by the values of the calculated confidence intervals, considering a 95% confidence level.

Table 2 presents the scores of the domains from the quality of the life questionnaire per gender.

**Table 2:** Scores of the domains from the quality of life questionnaire World Health Organization Quality of Life-BREF (WHOOQOL-BREF) with 26 items per gender

Domains	Men	Women	p value
	M±1.96×D	M±1.96×SD	(Wilcoxon test)
Physical	70.1213±28.61	67.7185±28.49	0.117
Psychological	69.3288±27.19	66.1133±25.13	0.031
Social	68.3629±33.03	66.5530±32.54	0.439
Environment	62.2067±25.69	59.4002±22.30	0.000*
General	67.5181±23.71	64.9304±21.87	0.006

* p value smaller than 0.00000000001 at 95% confidence interval.

M: mean; SD: standard deviation.

Quality of life, in all its domains, correlated negatively with the number of shifts (p<0.05). On average, at 14 shifts or more, QL started to decrease (p <0.05). Therefore, the QL was lower when physicians reported a higher number of shifts, namely: psychological well-being (r=-0.149, p<0.001), social relationships (r=-0.135; p<0.001), living environment (r=-0.149, p<0.001) and physical health (r=-0.118, p<0.001).

In addition, some QL factors correlated with the following demographic variables: living environment with income (r=0.13, p<0.001), physical health and psychological well-being with age (r=-0.08 and 0.0, p<0.05, respectively).

The study showed that 44.9% (498) of the physicians were addicted to work, 54.9% (610) were not addicted to work and 0.2% (2) did not properly answer the questionnaire.

When correlating work addiction with QL, we systematically and consistently verified that a greater addition led to a lower QL perception, according to the correlations of addiction with specific factors of this measure: psychological well-being (r=-0, p<0.001), social relationships (r=-0.34, p<0.001), living environment (r=-0.34, p<0.001) and physical (r=-0.32; p<0.001) and overall health (r=-0.43, p<0.001).

Table 3 shows the results of adjusting the chosen model after the four steps defined by the stepwise method.

**Table 3:** Estimated coefficients of variables selected by the stepwise method – model for quality of life

Variable	Coefficient estimate	V	p value	95%CI for OR
Age	-0.016	0.984	0.005	0.973-0.995
Gender	-0.410	0.664	0.003	0.506-0.870
Income	0.271	1.312	0.039	1.014-1.697
Number of shifts	-0.016	0.984	0.040	0.970-0.999
Work addiction (categories)	-1.289	0.276	0.000*	0.208-0.365

* p value smaller than 0.00000000001 at 95% confidence interval.

OR: odds ratio; 95%CI: 95% confidence interval.

Considering the 95% confidence interval, QL decreased as age increased (0.016 less chance of having high QL), lowering by up to 2.7% the chance of a high QL.

As for gender, since the reference category was male, there was evidence that being female contributed negatively to the QL (0.41 less chance of having high QL), lowering by up to 49.4% the chance of a high QL.

The higher the income, the higher the probability of physicians having a high QL (0.271 more chance of having high QL). Based on the respective odds ratios, an income increase could raise up to 1.697-fold the odds of a physician having high QL.

In respect to the number of shifts, more shifts implied a decrease in the physician’s chance of having a high QL (0.016 less chance of having high QL), which also revealed that the number of shifts was not the variable directly related with lower QL for physicians.

Since the reference category was non-addict, the opposite, *i.e.*, being an addict contributes negatively to the QL (1.289 less chance of having high QL), lowering by up to 79.2% the chance of a high QL.

Using the Hosmer and Lemeshow test, we found that the model was suitable to explain the outcome proposed, that is, high QL (χ^2^ of 2.706, degree of freedom=8, p=0.948), since the p value of the test was greater than 0.05. Moreover, the model had a general predictive power of approximately 67% of cases, which indicated a reasonable performance to explain the overall QL using the variables considered.

In view of the evidence that work addiction led to a decrease in QL, based on the χ^2^ test and the 95% confidence level, we observed an association between these variables, with a contingency coefficient of 0.274 (27.4%). We could say, therefore, there is a moderate association between both.

Since the non-high QL category was the reference, high QL contributed negatively to the possibility of a physician being more addicted to work (1.276 less chance of being addicted). We also verified that, when moving from the first (non-high QL) to the second category (high QL), the chance of a physician being more addicted to work decreased by up to 78.5%.

After finding an association between work addiction and QL in the two models adopted, it was possible to come up with some descriptive measures associated to the original scores of one variable for each level of the other classified variable. Table 4 presents the statistics of interest for each case.

**Table 4:** Score of work addiction for each classification of quality of life and *vice-versa*

	Mean	Standard deviation
Quality of life (%)		
<70	2.5507	0.57019
>70	2.1489	0.55428
Work addiction (%)	Mean	SD
<2.5	70.2468	10.56227
>2.5	61.5083	11.34901

Based on the results and the Mann-Whitney test (p<0.0001), work addiction scores for the QL groups were statistically different, considering the 95% confidence interval. The work addiction score was higher in the group of physicians with QL under 70%. Likewise, when we looked at the QL scores for the defined work addiction categories, work addiction groups had, in fact, statistically different QL scores at the 95% level (p<0.0001).

## DISCUSSION

The physicians from the State of Paraíba showed high QL. This may be directly associated with relatively well-paid salaries, medical appointments and/or medical procedures, especially in a region with a lower cost of living, which allows for a favorable financial situation and, consequently, a more comfortable life. All physicians in the study had income equal to or greater than seven minimum wages, which demonstrates this correlation.

Paraíba is small state, devoid of large metropolitan areas, which also contributes to a good QL, since the distances traveled are smaller and there is not much traffic. This gives doctors the privilege of quick access, as well as more time with their families. They can even have meals at home when they are not on call, which is not usual among these professionals.

A study investigating the QL of 62 primary care physicians using the WHOQOL-BREF showed high scores in the physical, social relationships and psychological domains, in agreement with this study.^3^ Another survey with professionals in healthcare centers found the highest scores in the WHOQOL-BREF for the physical health domain (mean 70.49) and the lowest for environmental health (mean 56.94).^22^

The higher means found in the physical domain show physicians believe they are fit to perform daily activities, with sufficient energy, good mobility, quality of sleep and work capacity; and they have less pain and less drug dependence.^22^ However, when compared to the physical, psychological and social domains, the environmental domain had the lowest average and was associated with issues involving financial resources, leisure, security and availability for information needed in everyday life.

Regarding the association between QL domain scores and gender, there is enough evidence pointing to a difference between the QL scores of men and women for both domains: psychological and environmental, with women presenting the lowest QL scores. In addition, being a woman decreases the chances of having high QL.

Factors such as pregnancy, childbirth, breastfeeding, domestic chores, participation in the education of children, besides the medical work itself may be associated with lower psychological^22^ and environmental health, since as long as there is a double burden (domestic and professional work), some factors, such as leisure and availability, are less present in the lives of these female doctors. Added to these factors is the financial inequality between genders - also present in this professional field - with men earning higher salaries when compared to women.^23^

Some authors investigated the QL of young Chinese physicians and found that being female, low schooling, low wages and working long hours are factors associated with poor QL among these professionals.^24^

In this study, there was a negative correlation between QL and the number of shifts. Working long hours and the requirement to work on an on-call basis keep physicians away from their families, the comfort of their homes and activities that give them pleasure. This situation can potentially have a negative impact on their physical and mental health, to the point of also compromising social interaction, which significantly contributes to a decrease in QL.

A study with orthopedic surgeons showed that the higher the workload, the lower the QL, with respect to the psychological dimension. In the environmental domain, the higher the income, the better the QL.^25^ Their findings corroborate those found in this study.

In the present study, age had a negative correlation with physical health (p=-0.08) and positive with psychological well-being (p=0.07); in that, the higher the age of physicians, the lower their physical health and greater their psychological well-being. This suggests that older physicians have worse physical health, which leads them to cut down on the number of shifts. In turn, this contributes to greater psychological well-being. Therefore, younger professionals, despite having good physical health, have worse psychological well-being, possibly due to the long working hours that prevent them from enjoying leisure time. Furthermore, the hospital itself is a stressful environment.

We observed that the more addicted to work, the greater the deterioration of the QL, which proves that work addiction can affect the QL of physicians. Workaholic professionals, by working excessively and compulsively, end up forgetting about the other domains in life (social, physical and mental), which are equally important for the maintenance of health and thus impair QL. In view of these findings, healthcare organizations must pay special attention to work addicts, since this behavior not only compromises one’s sociability with their work team, family life and health, but also decreases productivity, increases absenteeism and sick leaves, affecting the quality of the services provided.

Thus, strategies to improve QL in companies are necessary and can provide workers with increased well-being and pleasure in the workplace.

Strategies to improve QL are essential for the development and success of an organization and can make the work environment healthier and more enjoyable for employees. It can also favor more communicative and integrated teams and improve the health conditions of employees,^26^ which contributes to improving QL.

Companies and their managers need to identify the needs of their staff, so that labor improvements can be implemented in order to increase productivity and quality of care, as well as to solve or prevent situations that could compromise the well-being and health of employees.^26^ The implementation of QL strategies in the medical workplace, besides increasing employee satisfaction, can contribute to reducing accidents and errors, and improve patient care, since low QL directly affects the quality of care provided.

## CONCLUSION

The physicians participating in the research had good quality of life, with statistical significance in all domains. Quality of life can be affected by demographic characteristics such as age, gender and income. The number of shifts, in turn, correlated negatively with the quality of life, *i.e.* the higher the number of shifts, the worse the physician’s quality of life.

The data analyzed also showed that the worse a physician’s addiction, the more impaired is their quality of life. It seems clear that work addiction can affect the quality of life of physicians and contribute to its deterioration.

This study hopes to have contributed with new practices by shedding some light on the negative implications of work addiction on the quality of life of physicians, in order to alert the scientific community about this problem. We believe these results can give rise to proposals for interventions or strategies to improve the health and quality of life of medical professionals, thus improving the assistance provided in the public service.
